# Painless Operation

**Published:** 1847-07

**Authors:** 


### FACTS FROM CORRESPONDENTS.

### PAINLESS OPERATION.

Dr. J. T. Cogley, of Madison, in a letter of June 3d, mentions the following case:

A female, aged fourteen years, received a severe contusion from the falling of a house, in a storm, on the first of May. Gangrene, which was the result of the combined influence of the contusion and the obliteration of both the tibial arteries, commenced in the toes. At the end of two weeks from the time of the accident, a distinct line of demarcation had formed, an inch above the ankle joint. As there was great prostration and great constitutional disturbance, I desired to postpone amputation until there was an improvement in the general health; but a day or two after I thought the mortification had ceased to extend upwards, I discovered that the leg felt crepitous nearly as high as the knee. Dr. Rodgers being in consultation, we concluded to amputate immediately. Although the limb was exceedingly œdematous as well as crepitous, there was no appearance of gangrene *in the integuments* higher than I have specified; and on account of the importance of saving the knee joint, we decided to amputate about the middle of the leg.

The patient, having been placed upon the table ready for the operation, inhaled the ether for about two minutes, when the pulse became very frequent and feeble, and she appeared to have fallen asleep. I took hold of the leg and was about to cut, when she appeared to awake. The ether was again administered, and in two or three minutes she became perfectly unconscious.

I had determined to perform the flap operation, and commenced the incision on the outside of the fibula, carrying the knife around to the inside of the tibia in a semicircular direction. This anterior incision was nearly completed before the patient evinced the slightest appearance of consciousness. As I was about to insert the point of the knife for transfixion, she awoke, and while the knife was passing behind the bones and cutting out the flap, she evinced the ordinary symptoms of pain in such cases, but did not speak until I commenced sawing; then she inquired what we were doing. Ever since, she assures us that she was perfectly unaware of that part of the operation which preceded the sawing of the bone.

The condition of the posterior flap is worthy of record: All the cellular tissue in it was in a state of *suppuration and gangrene.*— When I saw the condition of the knee, I thought I should have to cut above the knee; but after a moment's consultation, we concluded to dress it, fearing that she would not bear the shock of a second operation,* and believing that in all probability she would die, cut where we would. I therefore pressed out all the pus, (which was black and had the characteristic odor of gangrene,) and injected a strong solution (ten grains to the ounce) of nitrate of silver, to which I added a few drops of pure nitric acid. We dressed with adhesive strips; no suture. At the first dressing subsequent to the amputation, some pieces of dead muscle came away, but the wound has been rapidly filling up with new granulations. It will be well in a few days, and there will be an excellent stump, with plenty of muscle over the end of the bones. There were no unpleasant symptoms produced by the ether: the operation was not followed by any reaction, which is doubtless attributable to the ether.

The result in this case leads me to believe that surgeons are sometimes unnecessarily apprehensive of the extension of gangrene in the stump, and consequently that knee joints have been removed which might have been spared.

Madison, June 3, 1847.

---

*At this stage of the operation we were under the impression that the ether did not prevent suffering, except for a moment in the commencement.

